# Association Between Hippocampus, Thalamus, and Caudate in Mild Cognitive Impairment APOEε4 Carriers: A Structural Covariance MRI Study

**DOI:** 10.3389/fneur.2019.01303

**Published:** 2019-12-20

**Authors:** Fabiana Novellino, María Eugenia López, Maria Grazia Vaccaro, Yus Miguel, María Luisa Delgado, Fernando Maestu

**Affiliations:** ^1^Neuroimaging Research Unit, Institute of Bioimaging and Molecular Physiology, National Research Council, Catanzaro, Italy; ^2^Department of Experimental Psychology, Universidad Complutense de Madrid, Madrid, Spain; ^3^Laboratory of Cognitive and Computational Neuroscience, Center for Biomedical Technology, Madrid, Spain; ^4^Networking Research Center on Bioengineering, Biomaterials and Nanomedicine (CIBER-BBN), Madrid, Spain; ^5^Institute of Neurology, University Magna Graecia, Catanzaro, Italy; ^6^Radiology Department, San Carlos Clinical Hospital, Madrid, Spain

**Keywords:** MCI, APOE, caudate nucleus, thalamus, structural covariance, MRI

## Abstract

**Objective:** Although, the apolipoprotein E (APOE) genotype is widely recognized as one of the most important risk factors for Alzheimer's disease (AD) development, the neural mechanisms by which the ε4 allele promotes the AD occurring remain under debate. The aim of this study was to evaluate neurobiological effects of the APOE-genotype on the pattern of the structural covariance in mild cognitive impairment (MCI) subjects.

**Methods:** We enrolled 95 MCI subjects and 49 healthy controls. According to APOE-genotype, MCI subjects were divided into three groups: APOEε4 non-carriers (MCIε4−/−, *n* = 55), APOEε4 heterozygous carriers (MCIε4+/−, *n* = 31), and APOEε4 homozygous carriers (MCIε4+/+, *n* = 9) while all controls were APOEε4 non-carriers. In order to explore their brain structural pattern, T1-weighted anatomical brain 1.5-T MRI scans were collected. A whole-brain voxel-based morphometry analysis was performed, and all significant regions (*p* < 0.05 family-wise error, whole brain) were selected as a region of interest for the structural covariance analysis. Moreover, in order to evaluate the progression of the disease, a clinical follow-up was performed for 2 years.

**Results:** The F-test showed in voxel-based morphometry analysis a strong overall difference among the groups in the middle frontal and temporal gyri and in the bilateral hippocampi, thalami, and parahippocampal gyri, with a grading in the atrophy in these latter three structures according to the following order: MCIε4+/+ > MCIε4+/− > MCIε4−/− > controls. Structural covariance analysis revealed a strong structural association between the left thalamus and the left caudate and between the right hippocampus and the left caudate (p < 0.05 family-wise error, whole brain) in the MCIε4 carrier groups (MCIε4+/+ > MCIε4+/−), whereas no significant associations were observed in MCIε4−/− subjects. Of note, the 38% of MCIs enrolled in this study developed AD within 2 years of follow-up.

**Conclusion:** This study improves the knowledge on neurobiological effect of APOE ε4 in early pathophysiological phenomena underlying the MCI-to-AD evolution, as our results demonstrate changes in the structural association between hippocampal formation and thalamo-striatal connections occurring in MCI ε4 carriers. Our results strongly support the role of subcortical structures in MCI ε4 carriers and open a clinical window on the role of these structures as early disease markers.

## Introduction

Individuals with mild cognitive impairment (MCI) have an increased risk of developing Alzheimer's disease (AD) compared to cognitively intact older people (1). There is a broad consensus that MCI subjects offer a potential model to understand factors involved in development of full-blown AD, before irreversible brain damage or mental decline has occurred.

Understanding neurobiological mechanisms happening in disease progression remains *a priori*ty of the scientific community, with the aim to create new intervention strategies for AD.

However, at present, the particular pathophysiological phenomena underlying the evolution across the MCI-to-AD continuum remain undetermined. Moreover, the risk factor action, which differently works in determining the pathophysiology of the disease, further adds complexity to the model.

One of the greatest risk factors is the apolipoprotein E (APOE) haplotype. The ε4 allele is present with a higher frequency in sporadic AD subjects than in the normal population (2, 3), and may interact with age at onset in modulating the clinical phenotype (4, 5). Indeed, the effect of the APOE ε4 genotype has been reported to be more deleterious in younger subjects, with accelerated rates of brain atrophy in regions particularly susceptible to deposition of neurofibrillary tangles and neuronal loss (6, 7). Less clear is how the APOE ε4 allele could act in modulating the expression of the disease, especially in early pathogenetic processes. Converging evidence from various pathological and *in vivo* studies suggests a region-specific effect of the ε4 allele on brain atrophy (8–13). Indeed, a gray matter (GM) loss selectively localized in the hippocampus, entorhinal cortex, and temporal pole (11), with relatively preserved volumes in the orbito-frontal cortex (11, 12, 14), has been reported in MCI– APOE ε4 carries. On the other hand, a growing body of evidence suggests that AD pathology propagates stepwise over time, following a specific topological pattern and involving large-scale brain networks (15, 16) rather than a focal brain region (17–19).

Based on the observation that related regions covary in morphometric characteristics, recent neuroimaging advances suggested that the study of anatomical structural covariance could represent a valuable tool to investigate the topological organization of the brain (20), providing complementary information to functional connectivity techniques. This approach was recently applied to AD subjects, providing critical support to the hypothesis that early disruptions in structural covariance between associative cortices and the entorhinal area could determine a disconnection potentially responsible to the clinical signs of AD (21). Moreover, a recent structural covariance study demonstrated that the pattern of structural association between the hippocampus and the rest of the brain differs as a function of APOE genotype in healthy young adults (22). This suggests that APOE genotype has an impact on topological organization of the brain.

Therefore, it could be hypothesized that differences in connectivity among regions differently affected by degenerative phenomena may underlie the different susceptibility to the development of AD in subjects with different APOE haplotype. At present, structural covariance approach has never been performed in subjects with MCI and different APOE genotype.

With the purpose to explore this hypothesis, the present study was designed to evaluate neurobiological effects of the APOE genotype in MCI subjects in terms of the degree of gray atrophy and structural covariance of specific brain areas differently affected, according to their APOE haplotype.

## Materials and Methods

### Subjects

Forty-nine healthy elders and 95 MCI patients were recruited from the Hospital Universitario San Carlos (Madrid, Spain) and from the Seniors Center of the District of Chamartín (Madrid, Spain). All subjects were right handed (23) and native Spanish speakers. Demographic and clinical data are shown in Table 1.

**Table 1:** Clinical and demographic characteristics in MCI patients and healthy controls.

	**MCI ε4−/− (*n* = 55)**	**MCI ε4+/− (*n* = 31)**	**MCI ε4+/+ (*n* = 9)**	**CTRL (*n* = 49)**	***p-*value**
Sex distribution (M/F)	21/34	11/20	4/5	15/34	0.801^*^
Age (mean ± SD)	74.1 ± 6.01	73.8 ± 4.1	74.6 ± 4.22	71.6 ± 4.26	0.076^◦^
Education years (mean ± SD)	9.33 ± 4.2	9.36 ± 4.87	9.43 ± 6.14	11.5 ± 4.17	0.088^◦^

* *Chi-square test*.

◦ *One-way ANOVA*.

*CTRL, controls; MCI, mild cognitive impairment; ϵ4–**/**–, APOEε4 non-carriers; ϵ4+/−, APOEϵ4 heterozygous carriers; ϵ4+/+, APOEϵ4 homozygous carriers*.

All participants were screened using the following standardized diagnostic instruments: The Mini Mental State Examination (24), the Global Deterioration Scale (25), and the functional assessment questionnaire (FAQ) (26). In addition, they also received an exhaustive neuropsychological assessment that included: Clock Drawing Test (27), Direct and Inverse Digit Span Test [Wechsler Memory Scale Revised (WMS-III)] (28), Immediate and Delayed Recall (Wechsler Memory Scale Revised) (28), Phonemic and Semantic Fluency (Controlled Oral Word Association Test) (29), Rule Shift Cards (Behavioral Assessment of the Dysexecutive Syndrome) (30), Visual Object and Space Perception Test (31), Boston Naming Test (32), and Trail Making Test parts A and B (33).

The MCI diagnosis was established according to the National Institute on Aging–Alzheimer Association criteria (34), which consists of the following: (i) self- or informant-reported cognitive complaints, (ii) objective evidence of impairment in one or more cognitive domains, (iii) preserved independence in functional abilities, and (iv) not demented (35). Besides meeting the clinical criteria, MCI participants exhibited a loss of hippocampal volume and therefore were categorized as “MCI due to AD” with an intermediate likelihood.

All participants were in good health, with no significant medical, neurological, or psychiatric diseases (other than MCI). General inclusion criteria considered an age between 65 and 85 years, with a normal MRI, without indication of infection, infarction, or focal lesions (rated by two independent experiences radiologists) (36, 37).

The 95 MCI subjects were classified according their APOE genotype: APOEε4 non-carriers (MCIε4−/−, *n* = 55), APOEε4 heterozygous carriers (MCIε4+/−, *n* = 31), and APOEε4 homozygous carriers (MCIε4+/+, *n* = 9). Due to the low frequency of the APOE ε4 genotype in the population of healthy controls (2), we limited the enrollment to healthy APOE ε4 non-carriers.

All MCI subjects underwent a clinical evaluation every 6 months for 2 years in order to evaluate their clinical progression.

The present study was approved by the Hospital Universitario San Carlos Ethics Committee (Madrid, Spain) in conformity to the Declaration of Helsinki, and all participants signed a written informed consent prior to their participation.

### MRI Acquisition

3D T1-weighted anatomical brain MRI scans were collected with a General Electric 1.5-T MRI scanner using a high-resolution antenna and a homogenization PURE filter (Fast Spoiled Gradient Echo sequence with the following parameters: repetition time/echo time/inversion time, 11.2/4.2/450 ms; flip angle, 12°; 1-mm slice thickness; a 256 × 256 matrix; and field of view, 25 cm).

### APOE Genotype

Genomic DNA was extracted from 10 ml blood samples in ethylenediaminetetraacetic acid. APOE haplotype was determined by analyzing single nucleotide polymorphisms rs7412 and rs429358 genotypes with TaqMan assays using an Applied Biosystems 7900 HT Fast Real Time PCR machine (Applied Biosystems, Foster City, CA). A genotyping call rate over 90% per plate, sample controls for each genotype, and negative sample controls were included in each assay. Three well-differentiated genotyping clusters for each single nucleotide polymorphism were required to validate results. Intra- and inter-plate duplicates of several DNA samples were included (38). According to the presence or absence of the ε4 allele, participants were classified as APOEε4 heterozygous (MCIε4+/−) and homozygous carriers (MCIε4+/+), or non-carriers (MCIε4−/−) (see Table 1).

### MRI Study Design and Statistical Analysis

First, we looked for structural differences between the MCIs and controls in GM by using voxel-based morphometry (VBM) in the whole brain, with no *a priori* region of interest (ROI). Then, we examined differences in network structural covariance. Those brain areas in which we found significant GM differences among groups by using VBM were selected as ROIs for structural covariance analysis. For both VBM and structural covariance approaches, we set the significance threshold at *p* < 0.05 with family-wise error (FWE) correction for multiple comparisons in the whole brain. Finally, with the aim to evaluate any association between clinical and neuropsychological variables and the significant clusters in both VBM and structural covariance analysis, we performed a Pearson's correlation analysis. For each subject, the mean values of GM intensity were extracted from each significant cluster in VBM analysis and then correlated with cognitive scores. To explore the relationship between structural covariance patterns and cognitive performance, individual brain scores for each significant cluster in the structural covariance analysis were extracted and then correlated with the neuropsychological scores. Correlational analysis was done separately for each group. Due to the number of tests conducted, the Bonferroni correction for multiple comparisons was calculated.

We evaluated differences among groups in the categorical variables (sex distribution, proportion of patients with disease progression) using the chi-square test. The Shapiro-Wilk test was used to check for normality before performing comparisons between continuous variables. Based on the results of this test, we used the one-way ANOVA, followed by the unpaired *t*-test corrected by Bonferroni, to assess differences among groups in age and education.

We used a multivariate analysis for neuropsychological scores. The assumption check for multivariate normality was tested through the Q-Q plot. Based on the results of this test, we performed the multivariate analysis of variance (MANOVA), followed by the Tukey's test for the *post-hoc* comparison. A series of MANOVAs were performed, one for each cognitive domain (general areas, memory, frontal/executive, language and visuospatial measures), using the group status (i.e., MCIε4−/− MCIε4+/−, MCIε4+/+, control) as the independent factor. Measures of effect size were expressed as partial eta square (η^2^) values. Statistical significance was set at *p* < 0.05.

### VBM

Data were processed and examined using the SPM8 software (Wellcome Trust Center for Neuroimaging, London, UK), running under MATLAB R2010b (The MathWorks, Inc.).

The structural images were pre-processed using the VBM8 toolbox (http://dbm.neuro.uni-jena.de/vbm.html). Default parameters incorporating the DARTEL toolbox were used to obtain a high-dimensional normalization protocol. Images were bias-corrected, tissue classified, and registered using linear (12-parameter affine) and non-linear transformations (warping), within a unified model. Subsequently, the warped GM segments were affine transformed into Montreal Neurological Institute (MNI) space and were scaled by the Jacobian determinants of the deformations (modulated GM volumes). Finally, the modulated volumes were smoothed with a Gaussian kernel of 8-mm full width at half maximum. The GM volume maps were statistically analyzed using the general linear model based on Gaussian random field theory.

The individual smoothed-normalized GM maps were entered into a second-level general linear model ANOVA to obtain SPM-F maps that investigated the main effect of group (i.e., MCIε4−/−, MCIε4–/+, MCIε4+/+, and the control group) and to detect morphological differences in GM among all groups.

To remove the variance percentage related to variables of non-interest that could interfere with group differences such as age, gender, years of education, and individual total intracranial volume; they were included in the model as covariates of non-interest.

### Structural Covariance Analysis

The regions significantly different among groups by using VBM were selected as ROIs for structural covariance analysis. To investigate the network structural covariance, regional GM volumes of each ROI were extracted from the pre-processed images. The MNI coordinates for the ROIs were defined using a threshold of *p* < 0.05 FWE (whole brain) using a 4-mm sphere centered on the MNI coordinates derived from VBM results (**Table 3**). Image processing was carried out based on a previous reported protocol (21).

Separate correlation analyses were performed by entering the extracted GM volumes from each ROI as a covariate of interest. The statistical model included covariates indicating each subject's gender, age, years of education, and intracranial volume values. First, the four groups were separately modeled in all of the analyses, in order to identify for each ROI which voxels expressed a positive correlation within each group. Then, we obtained correlation maps for each group that were thresholded at *p* < 0.05, corrected for FWE, and displayed on a standard brain template to allow qualitative comparisons between the groups. Furthermore, statistical contrasts were set to identify, for each ROI, voxels that expressed differences in the regression slopes among groups. We considered these differences in slopes as the differences in “structural association.” Specific ANOVA contrasts were established to map the voxels that expressed a different structural association strength among groups. The threshold for the resulting statistical parametric maps was established at a voxel-wise at *p* < 0.05 FWE-corrected for multiple comparisons. In addition, for a less conservative exploration of the results, we investigated whether additional regions resisted at a threshold of *p* < 0.001 uncorrected, >15 contiguous voxels.

## Results

### Clinical and Neuropsychological Findings

Demographical characteristics for each group, as well as the differences among groups, are shown in Table 1. Sex distribution, age at examination, and education level did not reached statistical significance among groups.

The detailed scores totalized in the complete neuropsychological battery as well as the MANOVAs (*F*-test, *p*-values, Wilk's lambda, and η^2^ values) are shown in Table 2.

**Table 2:** Neuropsychological scores in MCI patients and healthy controls.

	**MCI ε4−/− (*n* = 55)**	**MCI ε4+/− (*n* = 31)**	**MCI ε4+/+ (*n* = 9)**	**CTRL (*n* = 49)**	***F*-values**	***p*-value**	**η^2^**
**GENERAL AREAS**^**a**^							
MMSE (mean ± SD)	26.6 ± 2.56	26.8 ± 3.01	25.4 ± 1.27	29.3 ± 0.86	51.66	< 0.001^#^	0.33
GDS (mean ± SD)	3 ± 0	3 ± 0	3 ± 0	1.06 ± 0.31	100.56	< 0.001^#^	0.95
FAQ (mean ± SD)	2.09 ± 2.14	2.79 ± 3.28	4.4 ± 3.21	0.02 ± 0.014	11.18	< 0.001^#^	0.25
**MEMORY MEASURES**^**b**^							
Immediate recall (mean ± SD)	15.6 ± 9.72	14.8 ± 9.13	12.1 ± 5.37	39.4 ± 8.22	50.4	< 0.001^#^	0.61
Delayed recall (mean ± SD)	6.71 ± 7.3	4.48 ± 7.01	1.86 ± 3.34	25 ± 7.17	52.6	< 0.001^#^	0.62
Direct digit span (mean ± SD)	6.65 ± 1.73	6.69 ± 2.32	6.71 ± 1.8	9.40 ± 3.03	6.57	< 0.001^#^	0.17
Inverse digit span (mean ± SD)	4.21 ± 1.47	4.28 ± 1.2	3.86 ± 1.57	5.98 ± 2.2	5.89	< 0.001^#^	0.15
**FRONTAL/EXECUTIVE MEASURES**^**c**^							
Rule shift card (mean ± SD)	1.92 ± 1.34	2.19 ± 1.33	1.67 ± 1.21	3.21 ± 1.18	4.54	< 0.005^◦^	0.25
TMT-A (mean ± SD)	86.6 ± 36	80.3 ± 41.7	101 ± 17	51.7 ± 20.1	6.54	< 0.001^#^^,^^+^	0.17
TMT-B (mean ± SD)	235 ± 113	244 ± 117	333 ± 94.8	119 ± 58.8	13.08	< 0.001^#^^,^^§^	0.29
Phonemic Fluency (mean ± SD)	15 ± 4.44	7.98 ± 4.3	9.09 ± 3.92	9.99 ± 4.54	15.9	< 0.001^#^	0.31
**VISUO-SPATIAL MEASURES**^**d**^							
VOSP (mean ± SD)	6.58 ± 3.17	6.84 ± 3.35	6.86 ± 2.27	8.15 ± 3.9	0.89	0.512	0.027
Clock drawing test copy (mean ± SD)	6.91 ± 1.22	7.28 ± 2.29	6.40 ± 0.89	7.11 ± 1.22	1.34	0.262	0.041
**LANGUAGE MEASURES**^e^							
BNT (mean ± SD)	43.5 ± 9.55	47.7 ± 11.7	47.3 ± 5.91	55 ± 6.3	10.8	< 0.001^#^	0.25
Semantic Fluency (mean ± SD)	11.5 ± 3.57	13.2 ± 3.86	12.4 ± 2.15	16.9 ± 3.9	9.98	< 0.001^#^	0.23

a *MANOVA: Wilk's lambda = 0.038; F = 91.4; p < 0.001; η^2^ = 0.89*.

b *MANOVA: Wilk's lambda = 0.32; F = 15.8; p < 0.001; η^2^ = 0.31*.

c *MANOVA: Wilk's lambda = 0.58; F = 5.76; p < 0.001; η^2^ = 0.18*.

d *MANOVA: Wilk's lambda = 0.94; F = 1.25; p = 0.28; η^2^ = 0.025*.

e *MANOVA: Wilk's lambda = 0.66; F = 10.3; p < 0.001; η^2^ = 0.26*.

The significant differences in the Tuckey's post-hoc comparisons are reported below:

# *Significant differences in the post-hoc CTRL vs. MCI ϵ4–**/**–, CTRL vs. MCI ϵ4+/−, and CTRL vs. MCI ϵ4+/+ comparisons*.

◦ *Significant differences in the post-hoc CTRL vs. MCI ϵ4–**/**– and CTRL vs. MCI ϵ4+/+ comparisons*.

+ *Significant differences in the post-hoc MCI ϵ4–**/**– vs. MCI ϵ4+/+*.

§ *Significant differences in the post-hoc MCI ϵ4–**/**– vs. MCI ϵ4+/+ and MCI ϵ4+/− vs. MCI ϵ4+/+ comparisons*.

*CTRL, controls; MCI, mild cognitive impairment; ϵ4–**/**–, APOEϵ4 non-carriers; ϵ4+/−, APOEϵ4 heterozygous carriers; ϵ4+/+, APOEϵ4 homozygous carriers; BNT, Boston Naming Test; FAQ, Functional assessment questionnaire; GDS, Global Deterioration Scale; MMSE, Mini Mental State Examination; TMT-A, Trail Making Test part A; TMT-B, Trail Making Test part B; VOSP, Visual Object and Space Perception Test*.

MCI subjects showed significantly lower scores than did the controls in almost all cognitive tests, with the exception of clock drawing copy test and Visual Object and Space Perception Test. The *post-hoc* analyses revealed that the three MCI groups differed in attentional and executive function (Trail Making Test A: MCIε4−/− vs. MCIε4+/+, *p* = 0.038; Trail Making Test B: MCIε4−/− vs. MCIε4+/+, *p* = 0.024; MCIε4+/− vs. MCIε4+/+, *p* = 0.036), in which MCIε4 carriers performed worse (MCIε4+/+ > MCIε4+/−).

The clinical 2-year follow-up revealed that 36 out of 95 (about 38%) MCIs developed AD: 17 out of 56 (30%) patients in the MCIε4−/− group, 15 out of 32 (47%) in the MCIε4+/− group, and 3 out of 9 (34%) in the MCIε4+/+ group. The difference in the frequencies of patients who progressed to dementia was not statistically significant among the three MCI groups (χ^2^ = 2.66, *p* = 0.26).

### Differences Among Groups in VBM

**Figure 1:**
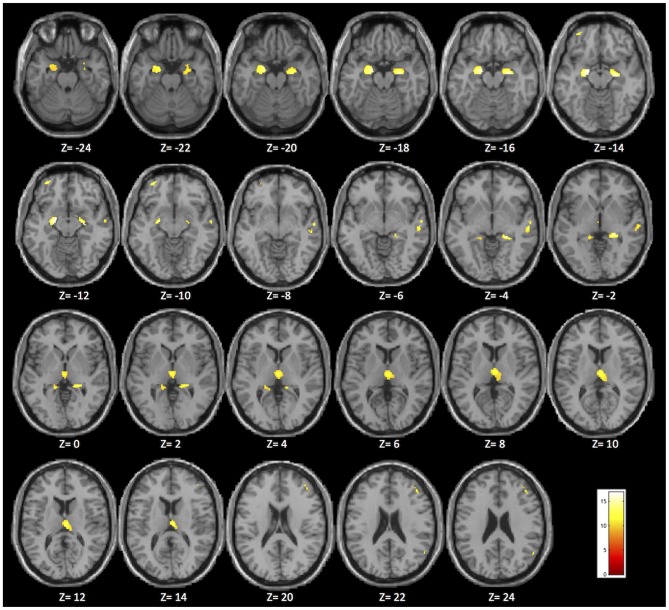
Brain areas in which significant differences were found among the MCIε4+/+, MCIε4+/−, MCIε4−/−, and control groups in the whole-brain voxel-based morphometry (VBM) analysis (*p* < 0.05 family-wise error (FWE) correction for multiple comparisons in the whole brain). The significant regions are superimposed on a standard template, with Montreal Neurological Institute (MNI) coordinates indicated at the bottom of each slice.

**Figure 2:**
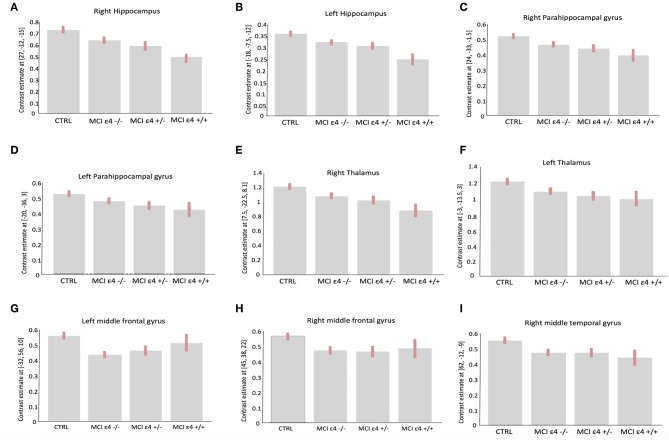
Plots depicting mean differences among groups within region of statistical significance in VBM analysis: right **(A)** and left **(B)** hippocampus; right **(C)** and left **(D)** parahippocampal gyrus; right **(E)** and left **(F)** thalamus, left **(G)** and right **(H)** middle frontal gyrus, and right middle temporal gyrus **(I)**.

Whole-brain VBM analysis revealed significant differences among groups in the following regions: right (x = −18; y = −7.5; z = −12) and left (x = 27; y = −12; z = −15) hippocampus, right (x = 8; y = −22.5; z = 8.1) and left (x = −3; y = −13.5; z = 3) thalamus, right (x = 24; y = −33; z = −1.5) and left (x = −20; y = −36; z = 3) parahippocampal gyrus, right middle frontal gyrus (x = 45; y = 38; z = 22), the orbital portion of the left middle frontal gyrus (x = −32; y = 56; z = −10), and right middle temporal gyrus (x = 62; y = −12; z = −9). The spatial extension and the significance of the single clusters are shown in Table 3 and in Figure 1. We found a grading in the atrophy of GM of the hippocampus, parahippocampal gyrus, and thalamus, ordered as follows: MCIε4+/+ > MCIε4+/− > MCIε4−/− > controls. The mean differences are shown in Figure 2. On the contrary, within the right and the left middle frontal gyrus, the GM atrophy observed in MCIε4−/− was more marked than the MCIε4+/− and MCIε4+/+ groups (Figure 2).

**Table 3:** Anatomical regions with significant differences among MCI ε4–**/**–, MCI ε4+/−, MCI ε4+/+ patients and control subjects in voxel-based morphometry analysis.

**Area**	Coordinates^*^			**Cluster extent**	***Z*-score**
	**x**	**y**	**z**		
Right hippocampus	−18	−7.5	−12	588	5.48
Left hippocampus	27	−12	−15	458	5.33
Right parahippocampal gyrus	24	−33	−1.5	217	5.05
Left parahippocampal gyrus	−20	−36	3	128	4.92
Right thalamus	8	−22.5	8.1	235	5.10
Left thalamus	−3	−13.5	3	221	5.11
Right middle frontal gyrus	45	38	22	61	5.04
Left middle frontal gyrus	−32	56	−10	64	5.43
Right middle temporal gyrus	62	−12	−9	107	5.33

*Significance was based on p < 0.05 family-wise error corrected*.

* *Coordinates are in Montreal Neurological Institute space*.

### Differences Among Groups in Structural Covariance Association

**Figure 3:**
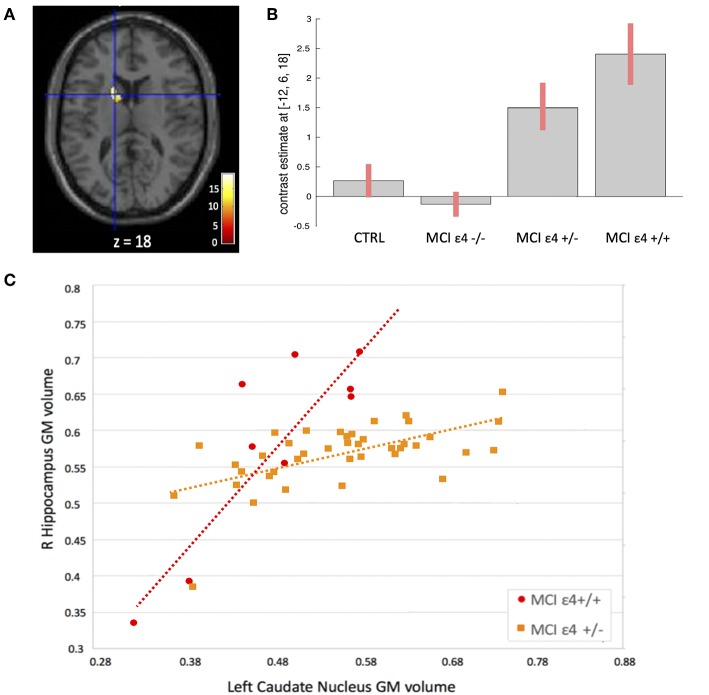
**(A)** Voxels that expressed the difference in structural association between the right hippocampus and the left caudate nucleus among the groups. **(B)** Plots depicting mean differences among the groups within region of statistical significance in structural covariance analysis. **(C)** Correlations between gray matter (GM) volumes extracted from 4-mm radius sphere centered on the ROI and the peak voxel expressing increased structural association in the MCIε4+/+ and in MCIε4+/− groups.

**Figure 4:**
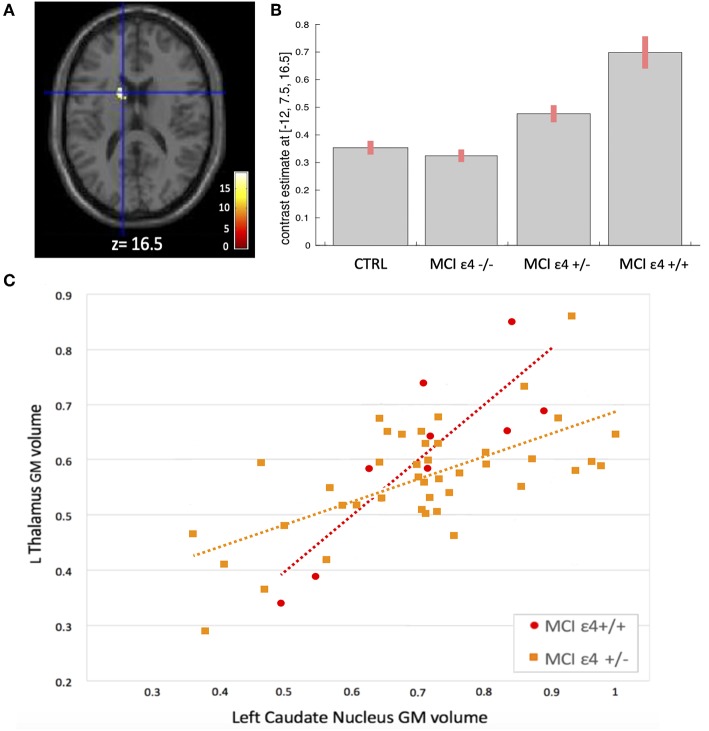
**(A)** Voxels that expressed the difference in structural association between the left thalamus and the left caudate nucleus among the groups. **(B)** Plots depicting mean differences among the groups within region of statistical significance in structural covariance analysis. **(C)** Correlations between GM volumes extracted from 4-mm radius sphere centered on the ROI and the peak voxel expressing increased structural association in the MCIε4+/+ and in MCIε4+/− groups.

We used seed-based structural covariance analyses to map the differences in pattern of structural covariance among the MCI (ε4–**/**–, ε4+/−, ε4+/+) and control groups. At the pre-established statistical threshold, an increased structural association between both the right hippocampus and the left caudate nucleus (x = −12; y = 6; z = 18; cluster size: 313), and the left thalamus and the left caudate nucleus (x = −12; y = 7.5; z = 16.5; cluster size: 203) was observed in both the MCIε4-carrier groups (Figures 3, 4; Supplementary Figures 1, 2). Moreover, this association was greater with increasing the dose of ε4 allele, being greater in homozygous (MCIε4+/+) than in heterozygous MCI carriers (MCIε4+/−). No significant differences, surviving at the statistical threshold, were obtained in the structural covariance starting from the other seed ROIs.

In addition, when we evaluated the results at a lower statistical threshold, we found differences among groups in the structural association between all the selected ROIs and a number of brain regions (highly recurring among the ROIs) including the caudate nucleus, fusiform gyrus, cingulum, precuneus, angular gyrus, superior and middle frontal, and temporal gyrus. The detailed results of differences among all groups with and without FWE correction are shown in Supplementary Tables 1–9.

### Relationship Between Clinical Findings and Structural Parameters

We performed regression analyses to investigate whether critical clinical and neuropsychological variables influenced the detected GM abnormalities in both VBM and structural covariance analysis.

Although present at the trend level, there were no significant correlations after the correction for multiple comparisons.

## Discussion

The present study investigated how APOE genotype modulated the whole-brain large-scale structural networks in MCI subjects. By using a double VBM and structural covariance mapping approach, we were able to demonstrate that MCIε4 carriers displayed a pronounced atrophy in specific regions including the thalamus and the hippocampus, and that both regions had strong structural covariance association with the left caudate nucleus. These findings suggest that the ε4 allele may affect the regional atrophy processes, resulting in a distinct picture in which the interaction among the hippocampus, thalamus, and caudate nucleus plays a peculiar and crucial role.

Our study demonstrates that there is a dose-dependent effect of the APOE genotype on the regional brain atrophy. Moreover, MCI ε4 carriers showed a pronounced degree of atrophy in the mesial temporal structures (hippocampus and parahippocampal gyrus), according to the dose of ε4 allele (MCIε4+/+ > MCIε4+/− > MCIε4−/−). On the contrary, MCI ε4 carriers had a relative preservation of the right and left middle frontal gyrus, in which the MCIε4−/− group showed the most pronounced changes. This evidence is in agreement with previous studies (11–14). Moreover, our results add the novel finding that also a subcortical structure, the thalamus, shared the same APOE dose-dependent effect with temporo-mesial structures, showing a more pronounced GM loss in MCIε4 homozygous carriers. However, the main novelty of this study was derived from structural covariance analysis showing a strong structural association between both the hippocampus and the thalamus (the regions with the most pronounced volume loss) and the caudate nucleus in ε4 carriers, mainly in MCIε4+/+ patients.

The results of this study provided interesting new insight into AD-related pathophysiology, pointing out the role of subcortical structures in MCI subjects carrying the APOEε4 allele, one of the most important risk factors for AD developing. These results raise some questions.

First, what is the meaning of the involvement of the subcortical structures in early AD pathophysiology.

In recent years, growing interest has been aroused by subcortical structures in AD, and a number of studies that underline the importance of these regions in early stages of the disease have been recently published. Convincing evidence is derived from studies conducted in AD mutation carriers in pre-symptomatic stages. Indeed, changes within the striatum and thalamus of these subjects have been reported throughout structural MRI studies (39–42) and functional PET experiments assessing the amyloid deposition (43–47). This demonstrates that the subcortical involvement in genetic AD is a peculiar and early phenomenon.

More challenging is to understand the role of thalamus and basal ganglia in sporadic AD.

Subcortical structures exhibit amyloid deposition in sporadic AD patients in pathological (15, 48) and *in vivo* PET studies (49, 50). This suggests a role of these regions also in this form of AD, although findings coming from MRI studies are not always univocal. Indeed, although the reduction of caudate and thalamus volumes has been previously described in the progression of MCI to AD (51–53), this evidence has not been found in another study, in which the putamen (but neither the thalamus nor the caudate nuclei) showed a volumetric loss in AD (54). Regarding the role of APOE genotype, interesting findings came from both structural and functional MRI studies. Changes in the caudate nucleus were found in APOE ε4 carriers compared with non-carriers healthy middle-aged adults (55), while intact APOE ε4 carriers showed reduced hippocampal connectivity with several brain regions (including the thalamus and caudate nuclei) compared with non-carriers (56). These findings (56) were related to the episodic memory performance.

In this debate, our results provide critical support to the hypothesis that the APOE genotype modulates the pattern of structural changes in MCI subjects. In particular, our findings suggest that in the ε4-carriers the caudate nucleus and the thalamus play a key role, along with the hippocampus, to trigger changes in the earliest stages of the disease. Our results indicate that, although pathophysiological model of AD has concentrated on cortical mechanisms, subcortical dysfunctions strongly contribute to the distinctive changes occurring in MCI ε4 carriers.

Another question regards the meaning of the intra-hemispheric structural correlation we found between the left thalamus and the left caudate and the inter-hemispheric between the right hippocampus and the left caudate.

The interaction between the ipsilateral thalamus and caudate has been broadly described in the context of the semantic memory retrieval circuit. This pathway, which include the cortical (mainly SMA) and subcortical (thalamus–caudate) circuitry, is engaged for complex, controlled semantic search and retrieval mechanisms (57). These operations include rule-based categorization, high-order categorization tasks, sentence comprehension with metaphor abstraction, category-driven word generation, and second language phonemic search (58–61). The left hemisphere in which we found the structural association can take on this interpretation given the prominent role of the left hemisphere in language skills.

The structural association we found between the right hippocampus and the left caudate indicates an inter-hemispheric interaction between these regions. This is quite surprising given that a large amount of studies described the ipsilateral interaction between the hippocampus and the caudate nucleus as critically involved in the map-based spatial memory, in navigation ability, and in decision making (62–64). However, due to the complexity in the hippocampal functional organization, the study of intra- and inter-hemispheric connections of hippocampus has aroused growing curiosity. A recent meta-analysis applied to high-resolution functional and structural neuroimaging studies demonstrated that the right anterior hippocampus had contralateral connection with a number of regions within large, distributed networks, including the head of caudate nucleus, and that these connections are engaged in high-order, complex task-processing (65). In addition, a study focused on the role of intrinsic hippocampal-caudate interaction in the human navigation, demonstrating that both ipsilateral and contralateral interactions showed significantly positive correlation with individual's navigation ability (66). Thus, the contralateral interaction between the hippocampus and the caudate we found could be interpreted in the context of these findings suggestive of a complex intra- and inter-hemispheric specialization of the hippocampus. This evidence is interesting also in light of the emerging pathogenetic hypothesis proposed for AD, which is now currently modeled as a disease that lead, since in the earliest stages, dysfunction within large-scale brain networks (21). The additional exploratory evaluation of the structural covariance we performed also shows results that go in this direction, revealing changes in structural association within large-scale brain areas (described in Supplementary Material), consistent with the default mode network (67) and the caudate–fusiform circuit (68).

Follow-up evaluation further heightens the strength of our results. Indeed, based on the clinical follow-up of the enrolled MCIs, we know that 36 out of the 95 patients (38%) developed AD after 2 years. Therefore, we can reasonably hypothesize that the structural changes we observed are early phenomena in the progression of the disease. The proportion of patients who progressed to dementia was not statistically different among the three MCI groups, although the frequency of converting subjects was higher in the MCIε4+/− group. This is probably due to the relatively short duration of the clinical follow-up, which in this study was 2 years. Although present at the trend level, no correlation survived the correction for multiple comparisons. This is probably a consequence of the rigorous statistical correction we have applied and to the small number of patients, especially the MCI homozygotes group.

It should be noted that this is a preliminary study, which may be interpreted in the context of some limitations. Although the initial sample size was large (95 MCI subjects), when we divided the MCI based on APOE genotype, the three groups were non-perfectly balanced by number, being the sample size of MCIε4+/+ smaller than the other groups. However, this is reasonable considering the different allelic frequency in the population, and our numbers are in line with the prevalence of allelic variants found in the Spanish population (69). We acknowledge that it would be important to replicate our findings in a larger sample. However, it is remarkable that, despite the smaller number of subjects in the MCIε4+/+ group, we could detect significant differences at stringent thresholds. Moreover, the clinical features were very homogeneous in terms of age, gender, years of education, and cognitive tests, thus increasing the reliability and the clinical relevance of our findings. Finally, the clinical follow-up of our patients to assess the rate of evolution to AD represents a strength of our work.

Since this is the first study demonstrating a specific pattern of structural covariance in MCI subjects according to their APOE haplotype, our results represent an interesting starting point for future studies. It will be interesting to explore the interaction between the hippocampus and subcortical structures through other structural approaches employing different morphometric methods (70, 71) as well as through structural and functional neuronal connectivity, inferred from diffusion tensor imaging and resting state-functional MRI (72, 73).

In conclusion, our study demonstrates *in vivo* that subcortical structures may be involved in APOE-related pathophysiological phenomena with a dose-dependent effect. Our study opens a new horizon on the role of these structures as potential early disease markers.

## Data Availability Statement

The datasets generated for this study are available on request to the corresponding author.

## Ethics Statement

The present study was approved by the Hospital Universitario San Carlos Ethics Committee (Madrid, Spain) in conformity to the Helsinki Declaration and all participants signed a written informed consent prior to their participation. The patients/participants provided their written informed consent to participate in this study.

## Author Contributions

FN contributed to the design of the study, drafting and revising the manuscript, and analysis and interpretation of the data. ML contributed to the data acquisition, draft writing, interpretation of results, and critical discussion of the manuscript. MV contributed to the analysis of the neuropsychological data, interpretation of results, and critical discussion of the manuscript. YM and MD contributed to the acquisition and analysis of the data and to the interpretation of results. FM contributed to the study concept/design, critical supervision of the article, and approval of the final version of the manuscript.

### Conflict of Interest

The authors declare that the research was conducted in the absence of any commercial or financial relationships that could be construed as a potential conflict of interest.
