# Supplementary material for: Association Between Hippocampus, Thalamus, and Caudate in Mild Cognitive Impairment APOEε4 Carriers: A Structural Covariance MRI Study
Source: Front Neurol. 2019 Dec 20;10:1303. doi: 10.3389/fneur.2019.01303 (PMC6933953; doi:10.3389/fneur.2019.01303)
Supplement: Supplementary file 1 [file Data_Sheet_1.PDF]

Supplementary Material

Supplementary table 1. Anatomical regions in which differences among MCI ε4-/-, MCI ε4+/-, MCI ε4+/+ patients and control subjects were found in structural covariance analysis with right hippocampus as seed.

Area	Coordinates*			Cluster extent	Z-score	F	peak-level°	
	x	y	z				p_{FWE}§	p_{uncorr}#
**Left caudate nucleus**	**-12**	**6**	**18**	**494**	**6.25**	**18.92**	**0.000**	**0.000**
Left fusiform gyrus	-24	-52	-15	747	4.30	9.55	0.066	0.000
Right calcarine cortex	22	-56	-16	489	4.29	9.54	0.068	0.000
Right medial superior frontal gyrus	4	46	30	373	4.21	9.24	0.091	0.000
Right anterior cingulum	9	46	9	210	4.01	8.52	0.187	0.000
Right caudate nucleus	15	14	12	351	3.92	8.21	0.250	0.000
Left anterior cingulum	-9	39	-6	355	3.91	8.17	0.258	0.000
Right superior temporal gyrus	60	-14	3	86	3.75	7.64	0.409	0.000
Right insula	33	10	4	106	3.75	7.64	0.411	0.000
Right superior frontal gyrus	24	12	58	34	3.74	7.61	0.418	0.000
Right fusiform gyrus	34	-44	-22	128	3.74	7.58	0.436	0.000
Right temporal pole	56	2	-18	214	3.71	7.53	0.446	0.000

°Both corrected and uncorrected results are reported
§ significance p<0.05 FWE (whole brain) corrected
# significance p<0.001 uncorrected, >15 contiguous voxels
*Coordinates are in MNI (Montreal Neurological Institute) space.

Supplementary table 2. Anatomical regions in which differences among MCI ε4-/-, MCI ε4+/-, MCI ε4+/+ patients and control subjects were found in structural covariance analysis with left hippocampus as seed.

Area	Coordinates*			Cluster extent	Z-score	F	peak-level°	
	x	y	z				p_{FWE}§	p_{uncorr}#
Left fusiform gyrus	-20	-51	-15	77	4.08	8.76	0.144	0.000
Right fusiform gyrus	26	-56	-16	44	3.54	6.98	0.644	0.000
Right angular gyrus	40	-64	33	39	3.49	6.85	0.694	0.000
Left caudate nucleus	-12	8	16	39	3.48	6.80	0.714	0.000

°Both corrected and uncorrected results are reported
§ Significance p<0.05 FWE corrected
# Significance p<0.001 uncorrected, >15 contiguous voxels
*Coordinates are in MNI (Montreal Neurological Institute) space.

Supplementary table 3. Anatomical regions in which differences among MCI ε4-/-, MCI ε4+/-, MCI ε4+/+ patients and control subjects were found in structural covariance analysis with right thalamus as seed.

Area	Coordinates*			Cluster extent	Z-score	F	peak-level°	
	x	y	z				p_{FWE}§	p_{uncorr}#
Left caudate nucleus	-12	12	9	71	3.52	6.94	0.665	0.000
Left middle frontal orbital gyrus	-30	50	-10	15	3.51	6.92	0.676	0.000
Right superior temporal gyrus	58	-10	-3	15	3.38	6.53	0.816	0.000
Right middle temporal gyrus	40	-60	14	17	3.36	6.47	0.837	0.000
Right caudate nucleus	14	10	14	40	3.28	6.18	0.914	0.000

°Both corrected and uncorrected results are reported
§ Significance p<0.05 FWE corrected
# Significance p<0.001 uncorrected, >15 contiguous voxels
*Coordinates are in MNI (Montreal Neurological Institute) space.

Supplementary table 4. Anatomical regions in which differences among MCI ε4-/-, MCI ε4+/-, MCI ε4+/+ patients and control subjects were found in structural covariance analysis with left thalamus as seed.

Area	Coordinates*			Cluster extent	Z-score	F	peak-level°	
	x	y	z				p_{FWE}§	p_{uncorr}#
**Left caudate nucleus**	**-12**	**7.5**	**16.5**	**474**	**5.49**	**14.75**	**0.000**	**0.000**
Right putamen	30	12	8	259	4.30	9.56	0.065	0.000
Left precuneus	0	-56	39	151	3.95	8.31	0.227	0.000
Right superior temporal gyrus	58	-9	-3	88	3.71	7.51	0.453	0.000
Right caudate nucleus	14	12	14	99	3.58	7.10	0.607	0.000
Left frontal middle orbital gyrus	-30	50	-10	18	3.54	7.00	0.648	0.000
Right rolandic operculus	58	2	-14	43	3.41	6.60	0.796	0.000

°Both corrected and uncorrected results are reported
§ Significance p<0.05 FWE corrected
# Significance p<0.001 uncorrected, >15 contiguous voxels
*Coordinates are in MNI (Montreal Neurological Institute) space.

Supplementary table 5. Anatomical regions in which differences among MCI ε4-/-, MCI ε4+/-, MCI ε4+/+ patients and control subjects were found in structural covariance analysis with right parahippocampal gyrus as seed.

Area	Coordinates*			Cluster extent	Z-score	F	peak-level°	
	x	y	z				p_{FWE}§	p_{uncorr}#
Right fusiform gyrus	22	-56	-15	88	3.45	6.72	0.741	0.000
Left anterior cinglulum	-9	39	-6	18	3.41	6.60	0.786	0.000
Right precuneus	12	-51	6	60	3.36	6.47	0.830	0.000
Left fusiform gyrus	-22	-52	-15	18	3.30	6.29	0.882	0.000

°Both corrected and uncorrected results are reported
§ Significance p<0.05 FWE corrected
# Significance p<0.001 uncorrected, >15 contiguous voxels
*Coordinates are in MNI (Montreal Neurological Institute) space.

Supplementary table 6. Anatomical regions in which differences among MCI ε4-/-, MCI ε4+/-, MCI ε4+/+ patients and control subjects were found in structural covariance analysis with left parahippocampal gyrus as seed.

Area	Coordinates*			Cluster extent	Z-score	F	peak-level°	
	x	y	z				p_{FWE}§	p_{uncorr}#
Left fusiform gyrus	-22	-48	-16	263	4.20	9.16	0.097	0.000
Right fusiform gyrus	24	-52	-17	276	4.06	8.69	0.155	0.000
Right precuneus	4	-57	48	71	3.71	7.53	0.440	0.000
Right anterior cingulum	14	32	27	75	3.60	7.18	0.570	0.000
Right cingulum	10	44	9	115	3.48	6.82	0.401	0.000

°Both corrected and uncorrected results are reported
§ Significance p<0.05 FWE corrected
# Significance p<0.001 uncorrected, >15 contiguous voxels
*Coordinates are in MNI (Montreal Neurological Institute) space.

Supplementary table 7. Anatomical regions in which differences among MCI ε4-/-, MCI ε4+/-, MCI ε4+/+ patients and control subjects were found in structural covariance analysis with right middle frontal gyrus as seed.

Area	Coordinates*			Cluster extent	Z-score	F	peak-level°	
	x	y	z				p_{FWE}§	p_{uncorr}#
Right SMA	10	18	52	432	4.02	8.12	0.096	0.000
Right precuneus	10	-57	34	236	4.01	8.06	0.212	0.000
Left middle frontal orbital gyrus	-10	52	-9	22	3.55	7.02	0.614	0.000
Left thalamus	-3	-21	0	54	3.50	6.87	0.670	0.000
Right cingulum	4	15	33	131	3.30	6.30	0.869	0.000
Right superior temporal pole	45	12	-21	15	3.23	6.10	0.917	0.001

°Both corrected and uncorrected results are reported
§ Significance p<0.05 FWE corrected
# Significance p<0.001 uncorrected, >15 contiguous voxels
*Coordinates are in MNI (Montreal Neurological Institute) space.
SMA: supplementary motor area

Supplementary table 8. Anatomical regions in which differences among MCI ε4-/-, MCI ε4+/-, MCI ε4+/+ patients and control subjects were found in structural covariance analysis with left middle frontal gyrus as seed.

Area	Coordinates*			Cluster extent	Z-score	F	peak-level°	
	x	y	z				p_{FWE}§	p_{uncorr}#
Left SMA	-14	3	64	81	3.79	7.78	0.346	0.000
Right postcentral gyrus	34	-36	51	28	3.62	7.25	0.524	0.000
Left caudate nucleus	-14	4	20	71	3.50	6.87	0.681	0.000
Left paracentral lobule	-8	-33	51	36	3.48	6.83	0.689	0.000

°Both corrected and uncorrected results are reported
§ Significance p<0.05 FWE corrected
# Significance p<0.001 uncorrected, >15 contiguous voxels
*Coordinates are in MNI (Montreal Neurological Institute) space.
SMA: supplementary motor area

Supplementary table 9. Anatomical regions in which differences among MCI ε4-/-, MCI ε4+/-, MCI ε4+/+ patients and control subjects were found in structural covariance analysis with right middle temporal gyrus as seed.

Area	Coordinates*			Cluster extent	Z-score	F	peak-level°	
	x	y	z				p_{FWE}§	p_{uncorr}#
Left fusiform gyrus	-20	-48	-14	354	4.07	8.74	0.156	0.000
Right parahippocampal gyrus	24	-42	-8	28	3.95	8.32	0.233	0.000
Right fusiform gyrus	20	-63	-15	159	3.86	8.00	0.312	0.000
Right superior medial frontal cortex	3	48	30	39	3.69	7.45	0.487	0.000
Left caudate	-16	4	18	126	3.67	7.38	0.514	0.000

°Both corrected and uncorrected results are reported
§ significance p<0.05 FWE corrected
# significance p<0.001 uncorrected, >15 contiguous voxels
*Coordinates are in MNI (Montreal Neurological Institute) space.

Supplementary Figure 1

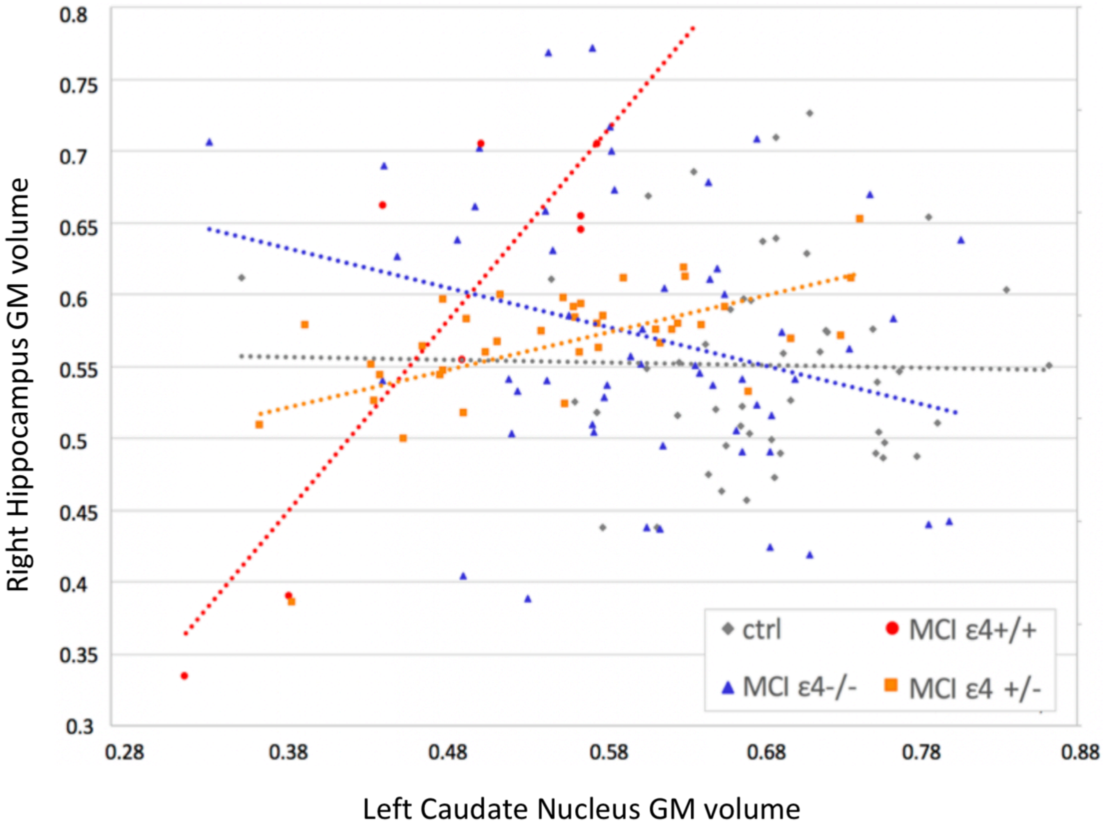

Legend to the Supplementary Figure 1: Correlations between GM volumes extracted from 4-mm radius sphere centered on the right hippocampus (seed region) and the peak voxel expressing structural association in all groups.

Supplementary Figure 2

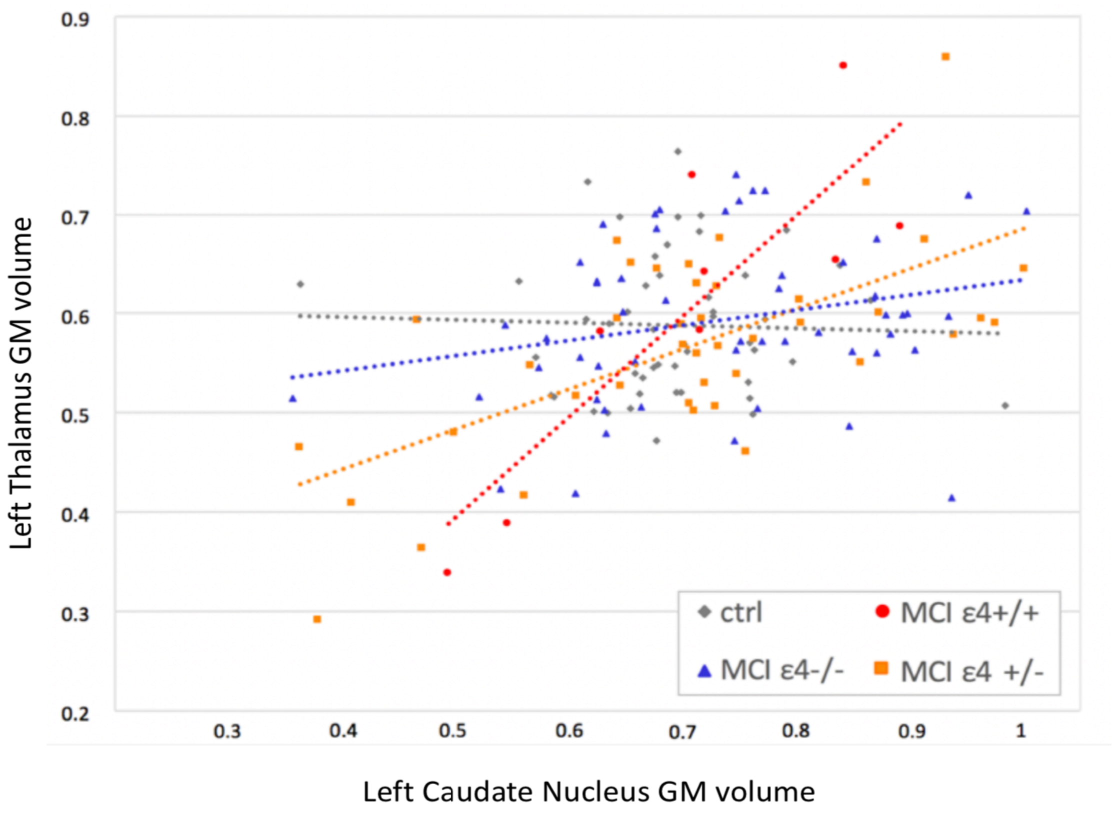

Legend to the Supplementary Figure 2: Correlations between GM volumes extracted from 4-mm radius sphere centered on the left thalamus (seed region) and the peak voxel expressing structural association in all groups.
